# IFNγ-Induced Bcl3, PD-L1 and IL-8 Signaling in Ovarian Cancer: Mechanisms and Clinical Significance

**DOI:** 10.3390/cancers16152676

**Published:** 2024-07-27

**Authors:** Suprataptha U. Reddy, Fatema Zohra Sadia, Ales Vancura, Ivana Vancurova

**Affiliations:** Department of Biological Sciences, St. John’s University, New York, NY 11439, USA; suprataptha.ummadi22@my.stjohns.edu (S.U.R.); fatema.sadia24@my.stjohns.edu (F.Z.S.); vancuraa@stjohns.edu (A.V.)

**Keywords:** IFNγ, Bcl3, PD-L1, IL-8, immune checkpoint blockade, ovarian cancer, cancer immunotherapies, tumor microenvironment

## Abstract

**Simple Summary:**

IFNγ is a multifunctional cytokine produced not only by activated lymphocytes but also in response to cancer immunotherapies; it has both antitumor and tumor-promoting effects. In ovarian cancer cells, the tumor-promoting effects of IFNγ are mediated by increased expression of the proto-oncogene Bcl3, the immune checkpoint PD-L1 and the proinflammatory cytokine IL-8. Recent studies have shown that IFNγ induces the expression of Bcl3, which then promotes the expression of PD-L1 and IL-8 in ovarian cancer cells, resulting in their increased proliferation and migration. In this review, we summarize the recent findings on the IFNγ tumor-promoting functions and on the mechanisms by which IFNγ induces Bcl3, PD-L1 and IL-8 expression in cancer cells, with a special focus on ovarian cancer. We also highlight the importance of a better understanding of these mechanisms to optimize the development of combinatorial approaches in cancer immunotherapies.

**Abstract:**

IFNγ, a pleiotropic cytokine produced not only by activated lymphocytes but also in response to cancer immunotherapies, has both antitumor and tumor-promoting functions. In ovarian cancer (OC) cells, the tumor-promoting functions of IFNγ are mediated by IFNγ-induced expression of Bcl3, PD-L1 and IL-8/CXCL8, which have long been known to have critical cellular functions as a proto-oncogene, an immune checkpoint ligand and a chemoattractant, respectively. However, overwhelming evidence has demonstrated that these three genes have tumor-promoting roles far beyond their originally identified functions. These tumor-promoting mechanisms include increased cancer cell proliferation, invasion, angiogenesis, metastasis, resistance to chemotherapy and immune escape. Recent studies have shown that IFNγ-induced Bcl3, PD-L1 and IL-8 expression is regulated by the same JAK1/STAT1 signaling pathway: IFNγ induces the expression of Bcl3, which then promotes the expression of PD-L1 and IL-8 in OC cells, resulting in their increased proliferation and migration. In this review, we summarize the recent findings on how IFNγ affects the tumor microenvironment and promotes tumor progression, with a special focus on ovarian cancer and on Bcl3, PD-L1 and IL-8/CXCL8 signaling. We also discuss promising novel combinatorial strategies in clinical trials targeting Bcl3, PD-L1 and IL-8 to increase the effectiveness of cancer immunotherapies.

## 1. Introduction

Ovarian cancer (OC) is the most aggressive gynecologic cancer, with poor survival and high mortality rates. One of the key features of OC is its heterogeneity, partly explaining, together with the lack of specific symptoms and biomarkers for early detection, the lack of successful treatments and poor outcomes [1,2,3,4,5,6,7,8]. The current treatment strategy includes tumor reduction surgery followed by platinum and taxane-based chemotherapy. However, since most patients relapse and develop chemoresistance, new therapeutic strategies are urgently needed. Immunotherapies have shown great promise in treating a variety of cancers, but in OC, the results have been disappointing. Further studies are needed to better understand the nature of immune signaling in OC and develop more effective therapeutic approaches to increase the effectiveness of cancer immunotherapies.

**Figure 1:**
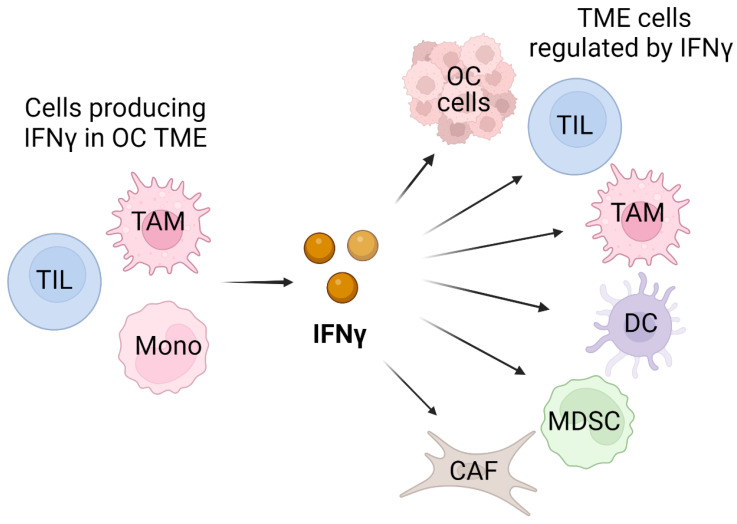
Schematic illustration of cellular sources and targets of IFNγ in OC TME. In addition to tumor-infiltrating lymphocytes (TILs), IFNγ can be produced by tumor-associated macrophages (TAMs) and monocytic cells (Monos) present in TME. The cellular targets of the produced IFNγ include OC cells and also TILs, TAMs, dendritic cells (DCs), myeloid-derived suppressor cells (MDSCs), as well as cancer-associated fibroblasts (CAFs). Created with BioRender.com with granted permission and license.

IFNγ is a pleiotropic cytokine that plays a major role in the immune response and cancer immunity [9,10,11,12,13]. In the tumor microenvironment (TME), IFNγ is produced predominantly by tumor-infiltrating lymphocytes (TILs), particularly by activated CD8 cytotoxic T cells. In addition, IFNγ expression is induced in response to immune checkpoint blockade (ICB) or radiation therapy used in cancer treatment [14,15,16,17,18]. However, since most OC tumors do not have high levels of TILs, other immune cells likely contribute to IFNγ expression in OC TME. In this regard, macrophages were reported to produce IFNγ [19], and since in OC, tumor-associated macrophages (TAMs) constitute over 50% of cells in the peritoneal tumor implants and the ascites [20], they likely represent another source of IFNγ. Furthermore, a study by Nowak et al. showed that peripheral blood mononuclear cells (PBMCs) isolated from OC patients produce appreciable levels of IFNγ [21], suggesting that mononuclear cells may also contribute to IFNγ expression in the TME. In addition to OC cells, IFNγ likely regulates other cells in the TME, including TILs, TAMs, dendritic cells (DCs), myeloid-derived suppressor cells (MDSCs), as well as cancer-associated fibroblasts (CAFs), thus having a central regulatory function in the TME (Figure 1).

Early studies revealed numerous antitumor effects of IFNγ, including enhanced MHC expression and antigen presentation on cancer cells, increased T-cell cytotoxic activity, and increased cancer cell cycle arrest and apoptosis [9,10,11,12,13,22,23,24,25,26,27,28,29]. Because of these antitumor properties, IFNγ has been used in cancer treatment [9,10,11,12,13]. However, most clinical trials using IFNγ have produced mixed or disappointing results [30,31,32,33,34]. Indeed, recent studies have shown that IFNγ also has important tumor-promoting functions that are less well understood and include the ability of IFNγ to induce the expression of immune checkpoints and T-cell exhaustion and increase cancer cell proliferation and migration [35,36,37,38,39,40,41,42,43,44]. In ovarian cancer, IFNγ induces the expression of the cancer antigen CA-125 (MUC-16) [45], human leukocyte antigen-E (HLA-E) [46], the immune checkpoint ligand PD-L1 [36,38,47,48,49], the proto-oncogene Bcl3 [47,50], and the pro-angiogenic and pro-inflammatory chemokine IL-8 [50,51], resulting in increased proliferation and migration of OC cells, immune escape, and tumor growth (Table 1).

Understanding the tumor-promoting mechanisms and cellular and molecular targets of IFNγ in ovarian cancer is crucial for minimizing its tumor-promoting functions in PD-1/PD-L1-blocking cancer immunotherapies that induce IFNγ expression and in other therapies associated with IFNγ release. Since Bcl3, PD-L1 and IL-8 promote tumorigenesis, combinatorial strategies targeting the IFNγ-induced Bcl3, PD-L1 and IL-8 expression in ovarian cancer may enhance the antitumor effects of cancer immunotherapies.

In this review, we summarize the current knowledge on the mechanisms by which IFNγ induces the expression of Bcl3, PD-L1 and IL-8/CXCL8 in cancer cells, with a special focus on ovarian cancer, and highlight the importance of a better understanding of these mechanisms to optimize the development of combinatorial approaches in cancer immunotherapies.

## 2. IFNγ Signaling in Ovarian Cancer

The biological effects of IFNγ are mediated by its binding to cell surface receptors IFNGR1 and IFNGR2, resulting in oligomerization of the receptor and phosphorylation and activation of the tyrosine Janus protein kinases JAK1 and JAK2 [9,52,53]. Activated JAK1/JAK2 then phosphorylate the transcription factor STAT1 at Tyr-701, resulting in its dimerization and nuclear translocation [52,53]. In addition to STAT1 phosphorylation at Tyr-701 by JAK1/2, STAT1 can be phosphorylated at Ser-727, which is required for its maximum transcriptional activity; however, the regulation of STAT1 phosphorylation on Ser-727 is cell specific [9,52]. In some cells, IFNγ signaling may also activate IκB kinase (IKK) to promote the expression of NFκB-dependent genes [54,55,56]. Furthermore, several studies have shown that IFNγ induces promoter histone acetylation by recruiting the histone acetyltransferases (HATs) p300 and CBP, resulting in increased promoter accessibility and transcription of interferon stimulated genes (ISGs) [57,58,59,60].

**Figure 2:**
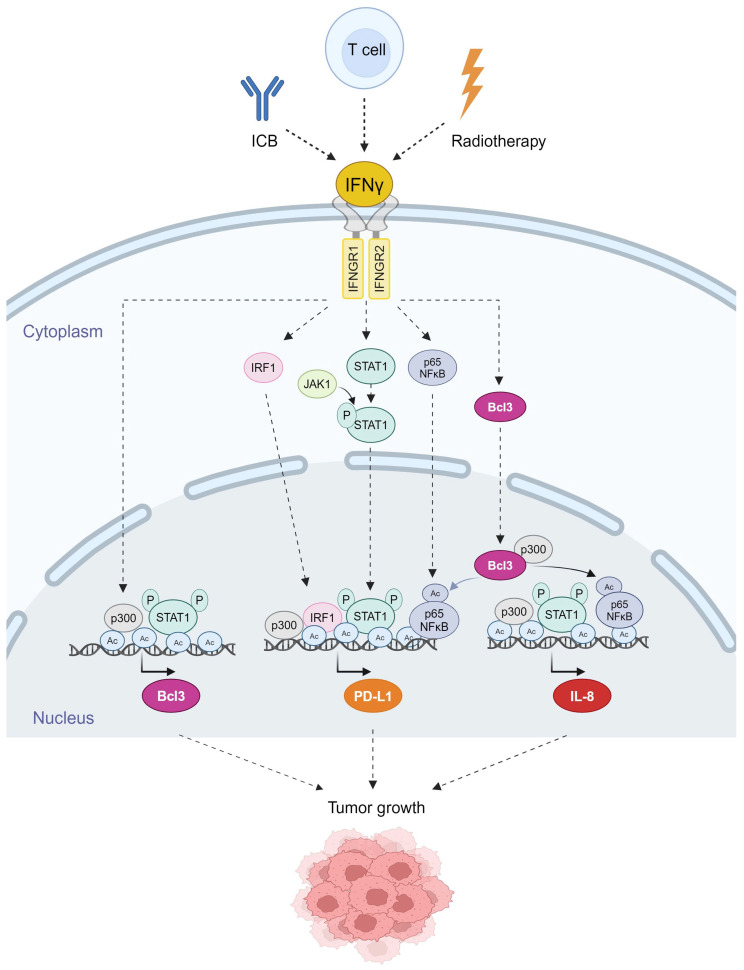
Schematic illustration of the mechanisms regulating IFNγ-induced Bcl3, PD-L1 and IL-8 expression in OC cells. IFNγ binds to its receptors IFNGR1 and IFNGR2, resulting in the activation of JAK1 kinase, and increased expression of the transcription regulators IRF1, STAT1, p65 NFκB and Bcl3 in OC cells [47,49,50]. JAK1 phosphorylates STAT1 at Tyr-701, which is required for the nuclear translocation of STAT1. STAT1 is then phosphorylated at Ser-727, which promotes its recruitment to the Bcl3, PD-L1 and IL-8 promoters. In addition, IFNγ induces p300-mediated acetylation of p65 NFκB at K314/315, resulting in increased p65 transcriptional activity and recruitment to PD-L1 and IL-8 promoters [47,49]. IFNγ also induces acetylation of histones (Ac) at the Bcl3, PD-L1 and IL-8 promoters in OC cells, thus facilitating transcription factor recruitment and transcription [47,48,49,50,51]. Created with BioRender.com with granted permission and license.

In ovarian cancer cells, IFNγ induces Bcl3, PD-L1 and IL-8 expression by mechanisms involving increased promoter acetylation, JAK1-STAT1 signaling, p65 NFκB signaling and Bcl3, resulting in increased OC cell proliferation and migration [47,48,49,50,51]; these mechanisms are schematically illustrated in Figure 2. In addition, a recent study has shown that IFNγ upregulates the expression of suppressor of cytokine signaling 1 (SOCS1) in OC cells, thus suppressing STAT3 and STAT5 and decreasing OC cell proliferation [61].

While early studies suggested that increased IFNγ levels correlate with improved clinical outcomes in patients with ovarian cancer [62], more recent studies have shown that high IFNγ expression in OC tissues promotes cancer progression and correlates with poor survival [36,46,63]. This discrepancy might reflect the high heterogeneity of OC tissues, different analytical methods used and the low stability of endogenous IFNγ in human serum and tissues.

## 3. Bcl3 in Ovarian Cancer

### 3.1. Bcl3 Signaling

The proto-oncogene and transcriptional regulator Bcl3 is a member of the IκB family of NFκB inhibitors, but unlike other IκB proteins, Bcl3 is localized predominantly in the nucleus. Since Bcl3 contains a transactivation domain, it can be recruited to NFκB-dependent promoters, resulting in transcriptional activation or repression, depending on the subunit composition of the NFκB complexes [64,65,66,67,68,69,70]. By binding to other NFκB proteins, Bcl3 regulates transcription of many NFκB-dependent genes, including genes involved in the regulation of the cell cycle, survival, proliferation, migration, chemotherapy resistance and cancer stemness, as well as pro- and anti-inflammatory genes [71,72,73,74,75,76,77,78,79,80,81]. In addition, Bcl3 can directly bind other transcriptional regulators, including histone acetyltransferases (HATs) and histone deacetylases (HDACs), thus regulating other signaling pathways [71,72,73].

Although Bcl3 was first identified in patients with chronic lymphocytic leukemia [64], its increased expression has been demonstrated in many types of hematological malignancies [82,83,84,85,86,87,88], as well as in solid tumors, including ovarian cancer, hepatocellular and nasopharyngeal carcinoma and colorectal, cervical, breast and prostate cancers [47,71,78,79,89,90,91,92,93].

### 3.2. Bcl3 Expression and Function in OC

Bcl3 expression is regulated by microRNA miR-125b, which is downregulated in OC tissues [94,95]. Overexpression of miR-125b decreases the Bcl3 levels in OC cells, resulting in decreased cell proliferation and cell cycle arrest and decreased tumorigenesis when injected into nude mice [94]. Bcl3 gene expression is increased in ovarian clear-cell adenocarcinoma, ovarian endometrioid adenocarcinoma, ovarian mucinous adenocarcinoma, ovarian serous adenocarcinoma, and ovarian serous surface papillary carcinoma [47]. Interestingly, compared to other OC types, ovarian serous surface papillary carcinoma has the highest levels of Bcl3 [47]; since tumors are often highly heterogeneous, it is plausible that a subset of OC cells might express considerably higher Bcl3 levels.

**Figure 3:**
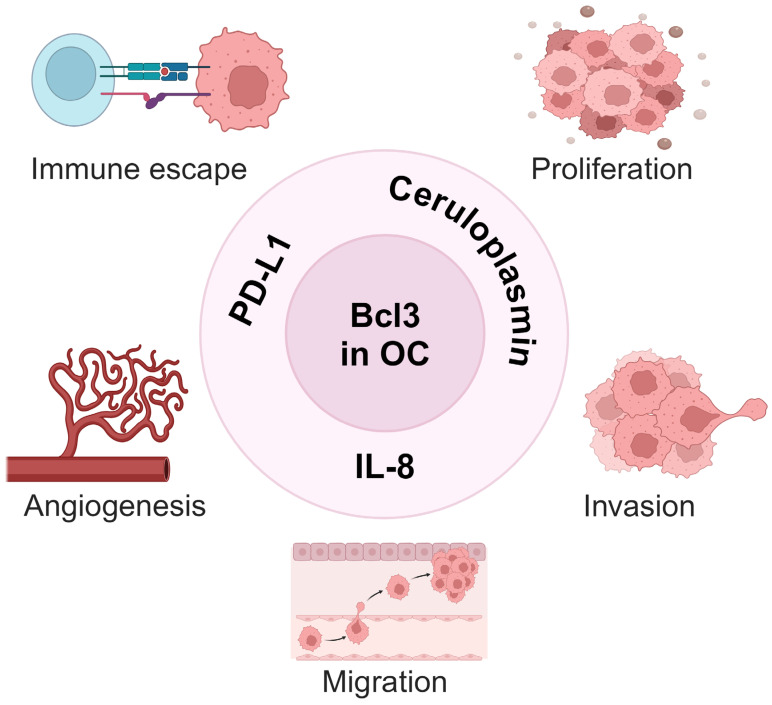
Bcl3 tumor-promoting functions in ovarian cancer. By inducing the expression of ceruloplasmin [96] and IL-8 [50,51], Bcl3 promotes proliferation, invasion and angiogenesis in OC cells. By inducing the expression of PD-L1 [47,49], Bcl3 also promotes immune escape in ovarian cancer. Created with BioRender.com.

In vitro studies demonstrated that Bcl3 suppression in OC cells induces their apoptosis and inhibits cell migration [47]. In addition, Dai et al. [96] showed that Bcl3 promotes survival, migration and invasion of OC cells by inducing the expression of the copper-carrying plasma protein ceruloplasmin. Intriguingly, we found that Bcl3 expression in OC cells is further enhanced by IFNγ, thus identifying Bcl3 as a new member of the ISGs [47,48,49,50]. IFNγ-induced Bcl3 facilitates the expression of IL-8 and PD-L1, linking the function of Bcl3 in OC to angiogenesis and immune escape [47,48,49,50,51]. Thus, the newly emerging functions of Bcl3 in ovarian cancer include the induction of OC cell proliferation, survival, migration and invasion and also angiogenesis and immune escape (Figure 3).

### 3.3. Mechanisms Regulating IFNγ-Induced Bcl3 Expression in OC

IFNγ-induced Bcl3 expression is facilitated by increased acetylation of the Bcl3 promoter and is dependent on both p65 NFκB and JAK1/STAT1 signaling [47,50] (Figure 2). IFNγ stimulation of OC cells increases acetylation of histones at the Bcl3 promoter, with a simultaneous promoter recruitment of Ser-727 pSTAT1, which is required for maximum transcriptional activity of STAT1 [50]. Although IFNγ-induced Bcl3 expression in OC cells is also dependent on p65 NFκB, p65 NFκB is not directly recruited to the Bcl3 promoter in OC cells [50]. However, p65 NFκB can mediate IFNγ-induced Bcl3 expression through binding to a downstream enhancer or through a cooperative interaction with STAT1. The latter scenario seems to be supported by several previous studies that reported crosstalk between NFκB and STAT1 in the regulation of IFNγ-induced inflammatory genes [9,97,98,99]. Future studies are needed to analyze in detail the mechanisms by which IFNγ induces the Bcl3 expression.

## 4. PD-L1 in Ovarian Cancer

### 4.1. PD-L1 Signaling

PD-L1 (also known as B7-H1 and CD274) was first identified as a transmembrane protein expressed on the surface of leukocytes, as well as non-lymphoid cells, including vascular endothelial cells in peripheral tissues [100,101,102,103]. By binding to PD-1 expressed on the surface of T cells, PD-L1 inhibits effector T-cell functions and immune responses. While the PD-1/PD-L1 pathway is central to maintaining immune tolerance and preventing autoimmunity, PD-L1 can also be expressed on tumor cells, thus attenuating tumor-specific immunity and promoting tumor growth [104,105,106,107,108,109]. PD-L1 expression in cancer cells is upregulated by IFNγ and other pro-inflammatory cytokines released by activated lymphocytes and other immune cells in the TME [36,38,39,40,41,104,105,106,107,108,109]. In addition, PD-L1 expression in cancer cells can be induced by IFNγ produced in response to chemotherapy, radiation or immune checkpoint blockade used in cancer treatment [10,110,111].

### 4.2. PD-L1 Expression and Function in OC

Immunotherapies blocking the PD-1/PD-L1 interaction have demonstrated great promise in treating a variety of cancers, but most patients fail to respond or develop resistance [41,104,105,112]. One of the factors determining the effectiveness of PD-1/PD-L1-blocking therapies is the surface expression of PD-L1. However, PD-L1 expression in tumors is highly heterogeneous, and varies among specific tumor types, cases, tissues and sampling times [36,104,112]. One plausible explanation for the high variability in PD-L1 in cancer tissues may be its dependence on the expression of IFNγ, which rapidly upregulates PD-L1 on most tumor cells [36,104,107,108,112]. In addition, the most widely used PD-L1 assay, immunohistochemistry, often measures mainly the surface levels of PD-L1 but not its intracellular levels; thus, additional mechanisms regulated by intracellular PD-L1 might be responsible for the limited responsiveness to PD-L1 targeted immunotherapies and the development of resistance.

Although PD-L1 was originally identified as a cell surface membrane protein [100,101,102,103], more recent studies have demonstrated its presence also in the cytoplasm and in the nucleus [113,114,115,116,117,118,119,120,121]. While the specific functions of intracellular PD-L1 are just emerging, several studies have shown that cytoplasmic PD-L1 acts as an RNA binding protein and regulates mRNA stability, while nuclear PDL1 can associate with DNA and regulate transcription [122,123,124,125]. In ovarian cancer, cell-intrinsic PD-L1 regulates cell proliferation, stemness gene expression, mTOR signaling, DNA damage response, autophagy and development of chemotherapy resistance [115,116,117,126,127,128,129].

High PD-L1 expression in OC tissues has been associated with poor outcomes; however, in contrast to other solid cancers, such as non-small-cell lung cancer (NSCLC), where PD-L1 expression has been used as a clinical indicator to select patients for anti-PD-1/PD-L1 therapy, in ovarian cancer, surface PD-L1 expression is not a good biomarker, and PD-1/PD-L1 blockade therapy does not significantly prolong progression-free survival [130,131,132,133,134,135,136,137,138,139]. Multiple causes are likely responsible for the limited effectiveness of PD-L1 as a clinical biomarker and therapeutic target in PD-L1 blocking therapies in OC; they may include the highly heterogeneous nature of OC tumors, the high inducibility and dependence of PD-L1 expression on IFNγ signaling and the incompletely understood functions of intracellular PD-L1, which is inaccessible to the currently used PD-1/PD-L1-blocking therapies. A better understanding of the mechanisms that regulate PD-L1 expression in OC cells and its intracellular functions should lead to the development of more effective PD-L1-targeting strategies for OC patients.

### 4.3. Mechanisms Regulating IFNγ-Induced PD-L1 Expression in OC

Although IFNγ is the main inducer of PD-L1 in most cells, the specific mechanisms and signaling pathways involved appear to be cell specific. The human PD-L1/CD274 promoter contains binding sites for the transcription factors NFκB, STAT1, IRF1 and hypoxia-inducible transcription factor (HIF) [47,140,141]. Consistent with the promoter multiple p65 NFκB binding sites, PD-L1 expression in OC cells treated with the chemotherapeutic drugs gemcitabine and paclitaxel is dependent on p65 NFκB signaling [110]. IFNγ induces the expression of p65 NFκB, its Bc3-facilitated acetylation and recruitment to PD-L1 promoter in OC cells [47,49] (Figure 2).

In addition, IFNγ-induced PD-L1 expression in OC cells is regulated by the canonical pathway, which involves the JAK1/STAT1 signaling [49]. IFNγ induces the expression of the transcription factor IRF1, which is then recruited to PD-L1 promoter and is required for maximum PD-L1 expression in OC cells [49]. Thus, IFNγ-induced PD-L1 expression in OC cells is dependent on simultaneous IFNγ-induced and p300-dependent PD-L1 promoter acetylation and promoter recruitment of the transcription factors IRF1, Ser-727-phosphorylated STAT1 and K314/315-acetylated p65 NFκB [44] (Figure 2).

## 5. IL-8 in Ovarian Cancer

### 5.1. IL-8 Signaling

Interleukin-8 (IL-8, CXCL8), which was originally discovered as a neutrophil chemoattractant and inducer of leukocyte-mediated inflammation, plays a crucial role in cancer progression through its induction of tumor cell proliferation, migration, invasion, angiogenesis and metastasis [142,143,144,145]. Furthermore, by binding to its receptors CXCR1/CXCR2, tumor-derived IL-8 recruits neutrophils (PMNs) and myeloid-derived suppressor cells (MDSCs), which play critical roles in tumorigenesis [146,147,148,149,150,151,152,153]. In contrast to the pro-inflammatory function of PMNs, the majority of recruited MDSCs are PMN-MDSCs with immunosuppressive activity [146,147,148,149,150,151,152,153]. In addition, the recruited PMNs and PMN-MDSCs release proteases that are associated with neutrophil extracellular traps and promote cancer progression [154]. IL-8 levels are increased in patients with advanced solid cancers, reflect the tumor burden and correlate with poor prognosis [155].

### 5.2. IL-8 Expression and Function in OC

IL-8 levels are increased in OC ascites, serum and tumor tissues, and increased IL-8 expression correlates with poor prognosis and survival in OC patients [156,157,158,159,160,161,162,163,164,165,166,167]. In addition, IL-8 polymorphisms have been associated with a greater risk of ovarian cancer [168]. IL-8 expression in OC cells is induced under stress conditions, such as hypoxia [157], acidic pH [169], the chemotherapeutic drug paclitaxel (Taxol) [170,171], the neurotransmitter hormone norepinephrine [172], proteasome inhibition [173,174], inhibition of histone deacetylation [175,176], surgical peritoneal stress [177] and fluid shear stress [178]. In addition, recent studies have shown that the IL-8 expression in OC cells is induced by IFNγ, adding IL-8 to the list of IFNγ-regulated genes [51].

**Figure 4:**
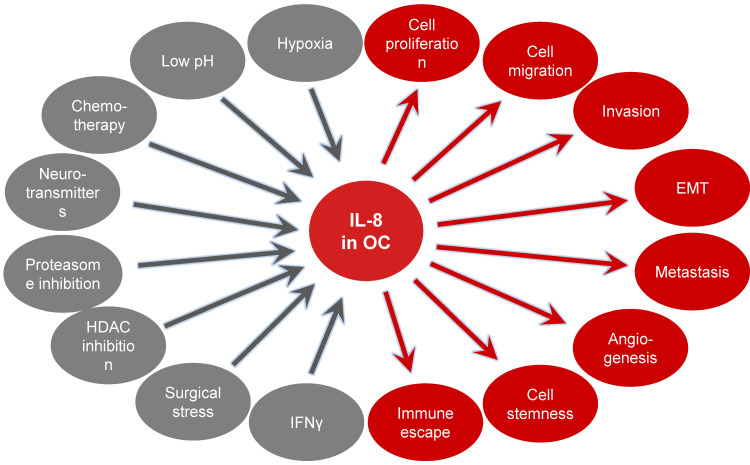
Mechanisms inducing IL-8 expression in ovarian cancer and the tumor promoting effects of IL-8 in OC cells.

Studies using human OC cells and tissues have shown that increased IL-8 expression enhances OC cell survival, proliferation, migration, and metastasis [50,51,179,180,181,182,183,184,185]. In addition, IL-8 increases OC growth by promoting epithelial-to-mesenchymal transition (EMT) [186,187,188], angiogenesis [189], cancer cell stemness [190] and immune evasion mediated by tumor-associated neutrophil infiltration [191] (Figure 4).

### 5.3. Mechanisms Regulating IFNγ-Induced IL-8 Expression in OC

Since mice do not have a homolog of the IL-8/CXCL8 gene, which is present in other species, including humans [192], compared to other cytokines, our understanding of the mechanisms that regulate IL-8 expression in cancer cells has been lagging. The IL-8 transcription is regulated predominantly by the transcription factor NFκB; in addition, in some cells, the transcription factors AP-1 and C/EBP are required for maximum IL-8 expression [193,194,195,196,197,198,199]. The human IL-8 promoter also contains a binding site for the transcription factor HIF-1α, which is adjacent to the NFκB site [199], consistent with hypoxia-induced IL-8 expression in OC cells [157].

In OC cells, IL-8 expression is regulated predominantly by p65 NFκB homodimers phosphorylated at serine 536 by IKK [173,174,175]. In addition, the IL-8 expression in OC cells is facilitated by increased acetylation of the IL-8 promoter and recruitment of IKK [175,176]. Inhibition of IKK suppresses IL-8 expression in vitro and increases the effectiveness of proteasome and HDAC inhibitors in vivo [175,176]. In this context, several studies have correlated the use of nonsteroidal anti-inflammatory drugs targeting the IKK enzymes with a reduced risk of ovarian cancer [200,201].

In IFNγ-stimulated OC cells, IL-8 expression is dependent on both JAK1/STAT1 and p65/Bcl3 signaling and is facilitated by concomitant promoter acetylation by p300 and recruitment of Ser-727 pSTAT1 and K314/315 ac-p65 NFκB (Figure 2). Neutralization of IFNγ-induced IL-8 using an anti-IL-8 blocking antibody reduces IFNγ-induced migration of OC cells and their invasion ability in 3D spheroids [51], indicating that IFNγ-induced IL-8 expression contributes to the pro-tumorigenic effects of IFNγ in ovarian cancer cells.

## 6. Bcl3, PD-L1 and IL-8 Co-Expression in Ovarian Cancer

### 6.1. Genomic Studies in OC

**Figure 5:**
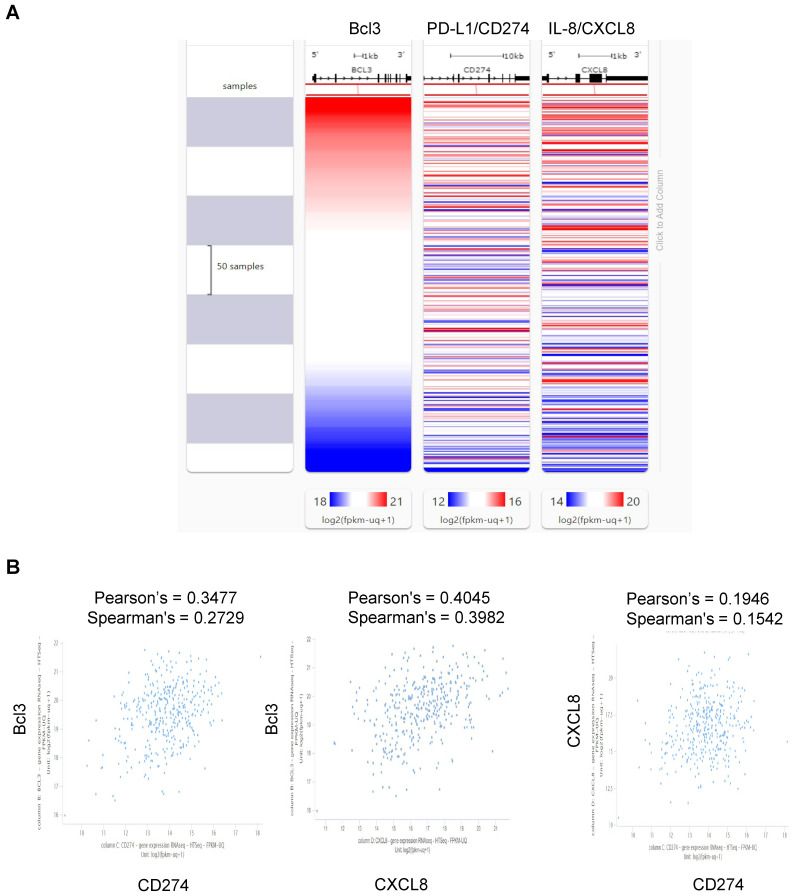
Bcl3, PD-L1 and IL-8 co-expression in ovarian cancer tissues. (**A**) Heatmap of Bcl3, PD-L1/CD274 and IL-8/CXCL8 mRNA co-expression in 379 OC samples in the GDC-TCGA database using the UCSC Xena platform. (**B**) Scatter plots showing associations between Bcl3 and PD-L1/CD274, between Bcl3 and IL-8/CXCL8 and between IL-8/CXCL8 and PD-L1/CD274 gene expression in GDC-TCGA ovarian cancer samples (*n* = 379) using the UCSC Xena browser.

Considering that the expression of Bcl3, PD-L1 and IL-8 in ovarian cancer cells is induced by IFNγ and dependent on JAK1/STAT1 signaling [47,48,49,50,51], we examined the gene co-expression profiles of Bcl3, PD-L1/CD274 and IL-8/CXCL8 in OC tissues using The Cancer Genome Atlas (TCGA) database and the UCSC Xena Browser (https://xena.ucsc.edu (accessed on 1 April 2024) [202]. Heatmap analysis of 379 OC samples from the GDC-TCGA database revealed a positive correlation between Bcl3, PD-L1/CD274 and IL-8/CXCL8 mRNA expression (Figure 5A). In addition, Pearson’s and Spearman’s correlation tests revealed positive correlations between Bcl3 and PD-L1/CD274, between Bcl3 and IL-8/CXCL8 and between IL-8/CXCL8 and PD-L1/CD274 expression in the GDC-TCGA database (Figure 5B). The positive correlation between IL-8 and PD-L1 in OC was also demonstrated by Wang et al., who showed that co-culture of ascites-derived ovarian cancer cells with CD8+ T cells resulted in increased gene and protein levels of IL-8 and PD-L1 in OC cells [203], and by Wu et al., who demonstrated IL-8 and PD-L1 co-expression in ovarian cancer organoids [204].

We have also examined the TCGA datasets using the cBioPortal for Cancer Genomics (https://www.cbioportal.org (accessed on 14 July 2024) [205] to evaluate whether there is any difference in overall survival (OS) in OC patients with altered expression of Bcl3, PD-L1 and IL-8. Analysis of 1949 samples revealed a statistical difference (*p* < 0.05) in OS in OC patients with altered Bcl3 expression but not PD-L1 or IL-8 expression; this is consistent with a model in which OS is driven mainly by the expression of Bcl3. However, more studies are needed to confirm these genome-wide studies also on the protein level.

### 6.2. Mechanisms Regulating Bcl3, PD-L1 and IL-8 Co-Expression in OC

**Figure 6:**
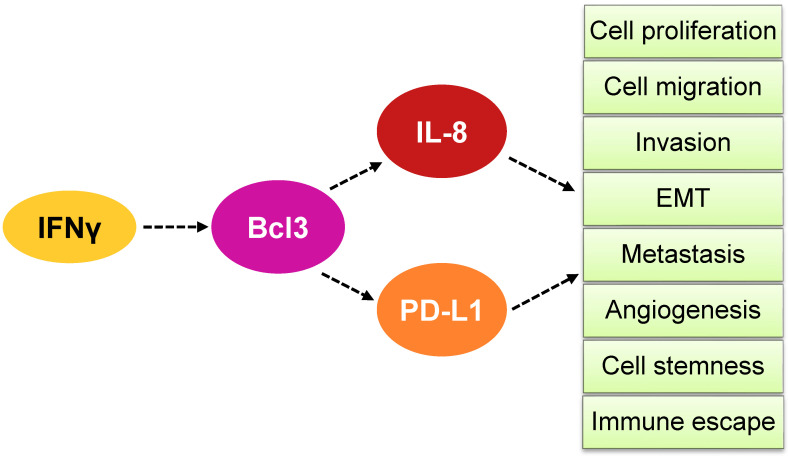
Model of how IFNγ induces Bcl3-dependent IL-8 and PD-L1 expression in OC cells. IFNγ induces first the expression of Bcl3 in OC cells, resulting in increased transcription of IL-8 and PD-L1. The increased expression of IL-8 and PD-L1 enhances OC cell proliferation, migration, invasion, epithelial-to-mesenchymal transition (EMT), metastasis, angiogenesis, cell stemness and immune escape.

The expression of Bcl3, PD-L1 and IL-8 in OC cells is induced by IFNγ; the underlying mechanisms involve the IFNγ-induced canonical JAK1/STAT1 pathway, as well as p65 NFκB signaling [47,48,49,50,51]. In addition, IFNγ enhances promoter acetylation, resulting in increased promoter recruitment and transcription of Bcl3, PD-L1 and IL-8. Interestingly, the proto-oncogene and transcriptional regulator Bcl3 is required for the maximum expression of both PD-L1 and IL-8 in IFNγ-stimulated OC cells [47,48,49,50,51] (Figure 2). These findings indicate that IFNγ first induces the expression of Bcl3, which then promotes the transcription of PD-L1 and IL-8 by facilitating promoter acetylation and p65 NFκB recruitment to PD-L1 and IL-8 promoters [47,48,49,50,51] (Figure 2). Since both PD-L1 and IL-8 induce proliferation, migration and invasion in OC cells, their IFNγ-induced expression likely contributes to the tumor-promoting effects of IFNγ in ovarian cancer (Figure 6).

Interestingly, several reports have indicated that IL-8 can directly upregulate PD-L1 expression in gastric, colorectal and esophageal cancer by mechanisms that include NFκB, p38 MAP kinase and mTOR signaling [206,207,208,209]. It will be important to determine whether IL-8 can also induce PD-L1 expression in OC cells and to identify the responsible mechanisms.

Furthermore, recent studies in other cancers have shown that increased systemic and tumor-associated IL-8 levels correlate with reduced clinical benefits of ICB therapies used in advanced melanoma, NSCLC, urothelial carcinoma, renal cell carcinoma, and glioma [210,211,212,213]. Since ICB therapies also enhance IFNγ expression [15,16,17,106], one of the underlying mechanisms may involve the IFNγ-induced IL-8 expression, resulting in increased cancer cell growth and resistance to ICB. These data suggest that targeting IL-8 signaling may increase the effectiveness of PD-L1-blocking therapies in ovarian cancer. To this end, future studies should determine whether immunotherapies targeting the PD-1/PD-L1 axis in OC patients increase their serum levels of IL-8.

## 7. Targeting IFNγ-Induced Bcl3, PD-L1 and IL-8 Expression

### 7.1. Targeting Bcl3 in OC

Bcl3 expression in OC cells promotes the expression of PD-L1 and IL-8, resulting in increased OC cell proliferation, migration, invasion and immune escape (Figure 6). Since Bcl3 gene expression is significantly increased in ovarian clear-cell adenocarcinoma, endometrioid adenocarcinoma, ovarian mucinous adenocarcinoma, ovarian serous adenocarcinoma and ovarian serous surface papillary carcinoma [47], Bcl3 might serve as a potential novel biomarker in OC. In this context, Bcl3 protein expression analyzed by immunohistochemistry is currently being tested as a potential biomarker to predict the response to alkylating chemotherapy in glioma patients (NCT03011671) [76,214]. Future studies should determine whether Bcl3 protein levels are increased in OC tissues and whether they correlate with PD-L1 and IL-8 expression.

In contrast to NFκB knockouts, Bcl3 knockout mice are viable, indicating that suppression of Bcl3 may represent a possible novel anticancer strategy [73,215]. Indeed, a recent study demonstrated that Bcl3-/- mice exhibit increased sensitivity to cisplatin chemotherapy in colorectal cancer, thus offering a rationale for targeting Bcl3 as an adjuvant to conventional therapies [216]. In this regard, Soukupova et al. recently developed small-molecule Bcl3 inhibitors with promising antimetastatic activity and low toxicity in triple-negative breast cancer [81]. Future studies are warranted to determine whether Bcl3 may serve as a potential biomarker in ovarian cancer and whether Bcl3 inhibition might be used as an antimetastatic strategy in ovarian cancer and other solid tumors.

### 7.2. Targeting PD-L1 in OC

In contrast to other solid tumors, such as NSCLC or melanoma, in ovarian cancer, surface PD-L1 expression has not been proven as a reliable biomarker to select patients for anti-PD-1/PD-L1 therapy and targeting the PD-1/PD-L1 signaling has produced disappointing results [106]. The underlying mechanisms likely include the highly heterogeneous nature of OC tumors, the high dependence of PD-L1 expression on IFNγ signaling, and the incompletely understood functions of intracellular PD-L1, which is inaccessible to currently used PD-1/PD-L1-blocking therapies. Since stability and intracellular localization and functions of PD-L1 are regulated by PD-L1 post-translational modifications that include acetylation and glycosylation, targeting these post-translational modifications may improve the effectiveness of PD-L1-targeting immunotherapies [123,217,218].

Interestingly, studies in melanoma, NSCLC, metastatic urothelial carcinoma and metastatic renal cell carcinoma have shown that resistance to PD-1/PD-L1-blocking immunotherapies correlates with increased serum levels of IL-8 [210,211,212,219,220], suggesting that IL-8 might serve as a biomarker predictive of resistance to PD-1/PD-L1-blocking therapies. Since IL-8 has multiple pro-tumorigenic effects, these studies have also indicated that suppressing IL-8 signaling might improve the outcomes of PD-L1-blocking therapies [210,211,212,219,220]. Indeed, numerous studies have evaluated the combination potential of PD-1/PD-L1-blocking immunotherapies with inhibiting IL-8 signaling, either by using small-molecule inhibitors that interfere with the IL-8 receptors CXCR1/CXCR2 or by using anti-IL-8 neutralizing antibodies [143,221,222,223].

### 7.3. Targeting IL-8 in OC

IL-8 signaling greatly influences the TME and promotes cancer progression by increasing cancer cell proliferation, angiogenesis and metastasis. In ovarian cancer, increased IL-8 expression induces proliferation and migration of OC cells, and increased IL-8 levels correlate with poor prognosis in OC patients [50,51,156,157,158,159,160,161,162,163,164,165,166,167,179,180,181,182,183,184,185]. In pre-clinical studies, blocking IL-8 signaling with the CXCR1/2 small-molecule inhibitors SX-682 or reparixin resulted in increased antitumor immunity and significantly slower tumor growth in gastric cancer [207], pancreatic cancer [224,225], squamous cell carcinoma [226], NSCLC [227], glioma [213] and hepatocellular carcinoma [228]. In addition, simultaneous inhibition of CXCR1/2 signaling by SX-682 and inhibition of TGFβ and PD-L1 signaling synergized to reduce mesenchymal tumor features in murine models of breast and lung cancer [229]. Blocking IL-8 expression with IL-8-neutralizing antibodies also suppressed the invasion of IFNγ-stimulated ovarian cancer cells grown in 3D spheroids [51]. Furthermore, blocking IL-8 signaling using an anti-IL-8 antibody resulted in decreased tumor growth and improved survival in a glioma mouse model treated with a PD-1-blocking antibody compared to PD-1 antibody alone [214].

HuMax-IL8 (BMS-986253) is a fully human monoclonal antibody that neutralizes IL-8 [230]. A phase I clinical study evaluated the safety and tolerability of HuMax-IL8, as well as changes in IL-8 serum levels in patients with incurable metastatic or unresectable solid tumors [230]. Although no objective tumor responses were observed, the antibody was safe and well-tolerated and associated with decreased serum IL-8 levels [230]. The acceptable safety profile of HuMax-IL8 (BMS-986253) suggested its potential in combination immunotherapies [230].

### 7.4. Clinical Studies Based on Simultaneous Inhibition of IL-8 and PD-1/PD-L1 Signaling

Several clinical trials have evaluated the use of combination immunotherapy based on simultaneous inhibition of PD-1/PD-L1 signaling and IL-8 signaling by using either the IL-8-neutralizing antibody HuMax-IL8 (BMS-986253) or blocking the IL-8 CXCR1/2 receptor. Ongoing clinical trials are evaluating the combination of HuMax-IL8 (BMS-986253) and PD-1/PD-L1-blocking immunotherapies in melanoma and advanced cancers (NCT03400332; NCT04572451), hepatocellular carcinoma (NCT04050462), prostate cancer (NCT03689699), pancreatic cancer (NCT02451982), head and neck squamous-cell carcinoma (NCT04848116), and colon carcinoma (NCT03026140) (Table 2).

In addition, there are ongoing clinical trials assessing the efficacy of PD-1/PD-L1-blocking antibodies and the small-molecule and orally bioavailable CXCR1/2 inhibitor SX-682 in treating melanoma (NCT03161431), metastatic colorectal cancer (NCT04599140; NCT06149481), pancreatic adenocarcinoma (NCT05604560; NCT04477343) and NSCLC (NCT05570825) (Table 2).

There have been no clinical trials evaluating the efficacy of combined inhibition of IL-8 and PD-1/PD-L1 signaling in ovarian cancer. However, considering that IL-8 expression is increased in OC tissues and that the neutralizing IL-8 antibody HuMax-IL8 (BMS-986253) and the CXCR1/2 inhibitor SX-682 are well tolerated, future clinical studies should assess whether the combination of IL-8 and PD-1/PD-L1 blockade might increase the effectiveness of PD-1/PD-L1-targeting immunotherapies in ovarian cancer patients.

## 8. Conclusions and Perspectives

Bcl3, PD-L1 and IL-8 have long been known to have important cellular functions as a proto-oncogene, an immune checkpoint ligand and a neutrophil chemoattractant, respectively. However, overwhelming evidence shows that these three genes have tumor-promoting functions far beyond their originally identified functions in many types of cancer, including ovarian cancer. These tumor-promoting mechanisms include increased cancer cell proliferation, migration, invasion, angiogenesis, EMT, metastasis, cancer cell stemness, resistance to chemotherapy and immune escape. The expression of Bcl3, PD-L1 and IL-8 is induced by IFNγ, which is produced not only by activated T cells but also in response to PD-1/PD-L1-blocking cancer immunotherapies. Perhaps not surprisingly, recent studies have shown that the IFNγ-induced Bcl3, PD-L1 and IL-8 expression is regulated by the same JAK1/STAT1 signaling pathway: IFNγ first induces the expression of Bcl3, which then promotes the expression of PD-L1 and IL-8 in ovarian cancer cells (Figure 6).

Since increased systemic and tumor-associated IL-8 levels correlate with reduced clinical benefit of PD-1/PD-L1-blocking therapies in solid cancers, including melanoma, NSCLC and glioma [210,211,212,213], serum IL-8 levels have been used as a biomarker for responsiveness to PD-1/PD-L1-targeting immunotherapies in those cancers. Based on recent in vitro studies in OC cells, future clinical studies are warranted to determine whether serum IL-8 levels may serve as a prognostic biomarker for PD-1/PD-L1-targeting therapies in ovarian cancer patients. In addition, considering the tumor-promoting functions of IL-8 and the low toxicity of the IL-8-neutralizing human antibody HuMax-IL8 (BMS-986253), blocking IL-8 signaling with IL-8-neutralizing antibody may increase the effectiveness of PD-1/PD-L1 immunotherapies in ovarian cancer patients.

As the expression of Bcl3, PD-L1 and IL-8 is induced by IFNγ, the expression of which greatly varies in cancer tissues, it will be important to analyze the expression levels of Bcl3, PD-L1, IL-8 and IFNγ in tumor tissues at the single-cell level. Future investigations should also determine whether immunotherapies targeting PD-1/PD-L1 signaling and other therapies associated with induced IFNγ release increase IL-8 serum levels and Bcl3 expression in tumor tissues.

Further studies are needed to better understand the nature of immune signaling in the ovarian cancer TME. There are many outstanding questions to be addressed: What are the specific cells, in addition to TILs, responsible for IFNγ production, and what are the cellular and molecular targets of IFNγ in OC TME? What are the specific mechanisms that regulate the stability and the transcriptional activity of Bcl3 and its ability to promote PD-L1 and IL-8 expression in OC? What are the specific functions of nuclear and cytoplasmic PD-L1 and what are the mechanisms that regulate the intracellular localization of PD-L1 and its stability? A more thorough understanding of these mechanisms will lead to the development of more reliable biomarkers and PD-1/PD-L1-targeting cancer immunotherapies. In addition, future clinical trials are warranted to assess whether the combination of IL-8 and PD-1/PD-L1 blockade might increase the effectiveness of PD-1/PD-L1-targeting immunotherapies in ovarian cancer patients.

## Figures and Tables

**Table 1:** Antitumor and tumorigenic functions of IFNγ in ovarian cancer cells.

IFNγ Antitumor Functions	IFNγ Tumorigenic Functions
Increased MHC expression [22,23]	Increased CA125 expression [45]
Increased T-cell migration [24]	Increased HLA-E expression [46]
Increased OC cell apoptosis [25,26]	Increased PD-L1 expression [36,38,47,48,49]
Decreased HER2 expression [27]	Increased Bcl3 expression [47,50]
Increased tumor cell lysis [28,29]	Increased IL-8 expression [50,51]

**Table 2:** Active clinical trials based on simultaneous inhibition of IL-8 and PD-1/PD-L1 signaling.

Reference	Combination Therapy	Disease	Phase
NCT03400332	BMS-986253 + Nivolumab	Melanoma and advanced cancers	I/II
NCT04572451	BMS-986253 + Nivolumab	Melanoma and advanced cancers	I
NCT04050462	BMS-986253 + Nivolumab	Hepatocellular carcinoma	II
NCT03689699	BMS-986253 + Nivolumab	Prostate cancer	I/II
NCT02451982	BMS-986253 + Nivolumab	Pancreatic cancer	II
NCT04848116	BMS-986253 + Nivolumab	Head and neck carcinoma	II
NCT03026140	BMS-986253 + Nivolumab	Colon carcinoma	II
NCT03161431	SX-682 + Pembrolizumab	Metastatic melanoma	I
NCT04599140	SX-682 + Nivolumab	Metastatic colorectal cancer	I/II
NCT06149481	SX-682 + Retifanlimab	Metastatic colorectal cancer	I/II
NCT05604560	SX-682 + Tislelizumab	Pancreatic adenocarcinoma	II
NCT04477343	SX-682 + Nivolumab	Pancreatic adenocarcinoma	I
NCT05570825	SX-682 + Pembrolizumab	Non-small-cell lung cancer	II

## Data Availability

All data are included in the article; further inquiries can be directed to the corresponding author.
